# The ethics of cosmetic overtreatment

**DOI:** 10.1016/j.jdin.2024.03.016

**Published:** 2024-04-04

**Authors:** Rachit Gupta, Joy Tao, David A. Hashemi, Roy G. Geronemus

**Affiliations:** a Division of Dermatology, Loyola University Medical Center, Maywood, Illinois; b Laser & Skin Surgery Center of New York, New York City, New York; c Ronald O. Perelman Department of Dermatology, New York University Langone Health, New York City, New York

**Keywords:** body dysmorphia, ethics, injectables, lasers, noninvasive cosmetic procedures, overtreatment

There has been a rapid increase in demand for cosmetic treatments in the United States, particularly noninvasive cosmetic procedures such as injectables, lasers, and related technologies.^1^ Elective cosmetic treatments are sought for a variety of reasons, including a desire for more youthful and rested appearance, more widespread acceptance of cosmetic procedures, and shifts in societal aesthetic preferences. However, in some cases, they may be pursued as a result of negative remarks from loved ones or psychiatric issues such as body dysmorphic disorder or addiction to cosmetic procedures.^2^^,^^3^ These factors may sometimes lead patients to request treatments that lead to severe distortion of their normal anatomy or “alienization.” Are cosmetic dermatologists obligated to treat patients asking for procedures that may appear unnatural or even harm the patient?

Patients who receive repeated cosmetic treatments may experience “perception drift,” which is a slow drift away from one’s original self-image. After a cosmetic treatment, patients develop a new baseline and subsequently fixate on new perceived flaws. As cosmetic treatments accumulate, a patient’s self-image can become altered.^4^ Social media has also contributed to unobtainable beauty standards that patients may try to achieve through serial cosmetic procedures, which may or may not fit their natural features.^1^

Cosmetic dermatologists have an obligation to do no harm (nonmaleficence) and avoid accepting payment or performing treatments that are unnecessary or actively harmful to patients. There is a myriad of potential risks associated with unnecessary cosmetic surgery, including poor aesthetic outcomes, scarring, burns, and other potential severe complications. In our experience, there has been a significant increase in the amount of corrective work we perform for patients, most commonly the consequence of procedures performed by poorly trained or unsupervised providers who may also accede to the unreasonable demands and expectations of their patients.^5^ Dermatologists must provide safe and effective treatments of the highest standard within their scope of training. Any dermatologist providing cosmetic treatment must be comfortable telling a patient “no,” even if this diminishes the physician-patient relationship or leads to patients seeking services elsewhere.

The patient may still insist on the requested cosmetic procedure, feeling enabled as this is an out-of-pocket expense; however, autonomy includes shared decision making with the physician. Before performing a treatment, several questions must be asked. Is the treatment likely to be beneficial? Will results lead to undesirable distortion of the patient’s anatomy? Is the treatment appropriate and safe for the patient’s skin type and condition? Do the potential benefits outweigh the risks of the procedure? Patients must be evaluated independently, and procedures should not be performed just because a previous provider performed it or because the patient is requesting it.

Although it may not seem like there is great benefit to denying care to a cosmetic patient, the denial contributes to dermatologists’ status and reputation as evidence-based physicians operating at the highest standard. Additionally, although a few patients may seek care elsewhere, saying “no” will help dermatologists build trust within the medical community as beacons of good judgment, practicing medicine in the best interest of their patients.

## Conflicts of interest

None disclosed.
